# 3-4-year-old children’s memory flexibility allows adaptation to an altered context

**DOI:** 10.1371/journal.pone.0275071

**Published:** 2022-09-23

**Authors:** Krisztina Liszkai-Peres, Dora Kampis, Ildikó Király

**Affiliations:** 1 Department of Ethology, Eötvös Loránd University, Budapest, Hungary; 2 Doctoral School of Psychology, Eötvös Loránd University, Budapest, Hungary; 3 Institute of Psychology, Eötvös Loránd University, Budapest, Hungary; 4 University of Copenhagen, Copenhagen, Denmark; 5 MTA-ELTE Momentum Social Minds Research Group, Psychology Institute, ELTE Eötvös Loránd University, Budapest, Hungary; 6 Cognitive Development Center, Central European University, Budapest, Hungary; University of Queensland, AUSTRALIA

## Abstract

Imitation provides a reliable method to investigate the developing memory functions in childhood. The present study explored whether 3-4-year-old children are able to revise their previous experiences after a 1 week delay in order to adapt to an altered context. We used a combined short-term (Session 1) and delayed (Session 2) imitation paradigm based on a previous study with 2-year-olds. The constraints (target object close/far) and relatedly the relevance of using a tool in a goal attainment task (irrelevant/relevant, respectively) changed between the sessions. We found that children in Session 1 used the tool only when it was needed (relevant/object far context). After the 1 week delay when the tool was previously irrelevant and then became relevant, children remembered the irrelevant act and applied it in the altered context. When the tool lost its relevance after 1 week, children used the tool less than before, but did not fully omit it, despite its reduced efficiency. The present data with 3-year-olds was compared to a pattern of results with 2-year-olds (from a similar previous study), that allowed to discuss possible developmental transitions in memory and imitation. We propose that the flexible restoration of a formerly irrelevant act and the maintenance of a formerly successful solution indicate flexibility of preschooler’s memory when guiding imitation. This flexibility, however, interacts with children’s tendency to remain faithful to strategies that were previously ostensively demonstrated to them.

## Introduction

In early childhood, imitation i.e., behavioral re-enactment is an important means for learning, both for instrumental purposes such as using a tool, and also for socially constituted forms of behavior, like gestures and even language [1, 2]. Already during the first year of life, infants are able to copy simple facial gestures and vocalizations ([3] but see [4]). With the development of the motor system, more complex behavior sequences are also imitated (for a review see [5]), and with age the length of the imitated sequence is constantly growing [6, 7]. Precision of imitation shows variability from high fidelity imitation, copying each action step, to goal emulation, when only the goal is copied, but the specific means leading to it are not [8–11]. This variability in the form of re-enactment highlights that the underlying mechanisms of imitation, in addition to direct copying [12] or automatic processes [13], include also one that is inferential in nature [14].

The notion that imitation is inferential is supported by the fact that goals play a modulatory role in what gets re-enacted. For example, children typically do not blindly copy a model’s unsuccessful action (”a failed attempt”); instead they imitate the model’s inferred goal with successful goal-attainment [14]. Children are also found to be selective in imitating a tool use, as they are more likely to imitate if the successful goal-attainment with a tool is preceded by another inefficient means [15, 16]. In addition, already at a young age, children tend to copy only those specific aspects of an event that they deem efficient in a given context [10]. In light of these results, the teleological account suggests that young children already apply the presumption that a goal is attained in the most efficient way possible within the constraints of a situation, and use this assumption in their choice of what to re-enact from the observed behavior–that is, they infer the intended goal of the action [17]. Though the teleological account has been challenged by low-level accounts of action interpretation (e.g., [12, 18, 19]), it could offer a viable solution for learning completely novel behaviors through goal identification and a related initiation of a search for a means to attain it (see [20]).

The inference-based nature of imitation makes it an excellent means to study the development of learning and memory in more depth [21, 22], by probing the understanding of causal relations and what gets retained from different events. Do irrelevant actions get filtered out? Do efficiency evaluations play a role in what is remembered? Memory studies applying delayed imitation paradigms, where some time passes between the demonstration of an action and the recall of this action (varying from 1 day to months), have probed the reliability and the capacity constraints of memory across development (e.g., [6]; for a review see [2, 21]). These studies do not examine, however, memory encoding and retrieval separately. Arguably, these two should be investigated separately to better understand what determines whether information is retained or filtered out at encoding, what part of said encoded information is retrieved after a delay, and what is filtered out at retrieval (or failed to be retrieved).

Memory is a dynamic system, in the sense that the same memory often has to be retrieved in various contexts and applied differently. Events in real life are often similar but not identical, and the solution to a previous problem can be transferred to another one with some correction, fitting to the new constraints. For example, if we have driven only a car with manual transmission, and then we have to drive a car with automatic transmission, we are still able to drive the car, but we have to tailor our behavior to the new circumstances. Evidence indicates that some foundations of such adjustment can be observed early on [23, 24]. For example, in the study of Williamson, Meltzoff and Markman [23] three-year-olds imitated a new opening method less if their own means had been efficient (easy opening context), compared to children with a difficult prior experience. Yet subsequently, if it became difficult to open the box, most children were able to retrieve the demonstrated act and use it instead of their now less efficient means [23]. Thus, three-year-old children were able to adjust their behavior relative to a past event (i.e., choose a different means they remember was demonstrated to them), based on the evaluation of how to reach the goal most efficiently.

Other times we have to adjust our present behavior in relation to a possible future event. In tasks involving episodic foresight, children have to prepare and overcome a possible future obstacle based on their previous experiences [25–27]. These studies show that even 2-year-olds show flexibility in a simpler future oriented task [28], and 3 and 4-year-olds could solve more complex tasks [26, 29]. However, in these paradigms the goal situations and the prior experience (the source of their memories) are usually close in time (ca. 5–30 minutes) (e.g., [26]). Additionally, most of these tasks do not require remembering the order of elements and thus potentially to understand the temporal-causal relations between the events in the original episode. Together, it remains an open question how memory flexibility works if the delay is longer than the interval with which short-term memory operates. Studying these processes with a longer delay between encoding and retrieval is essential to investigate what is consolidated and stored for the long-term in memory, and whether these memory traces are available for flexible adjustment in behavior.

Some studies found that the duration of delay changes the content of memories if retrieved by behavioral re-enactment [30, 31]. In Simpson and Riggs’ study [31], 3- and 4-year-old children imitated both irrelevant and relevant acts in the short-term, but after a week delay children mainly performed only the relevant actions in order to achieve a goal. Similarly, in a cross-cultural study by Kline et al. [30], children (between the age of 9 and 16 years) and adults imitated more precisely on the short-term, but omitted irrelevant actions after a one-month delay. Thus, it is possible that at the immediate recall all the elements were available in working memory and were retrieved from there, while for the long-term only the meaningful elements were stored and available for retrieval. This leaves open the question whether irrelevant elements could be retrieved if needed but are omitted due to situational demands, or whether long-term memory stores only the relevant action elements and the rest gets omitted during, or shortly after, encoding.

A study by Liszkai-Peres, Kampis and Király [32] investigated this question with 2-year-olds by changing the situational demands and the relevance of a tool use action in a short-term and long-term retrieval test. The study used two types of changes, which may require slightly different types of flexibility and adjustment. One type of change was that an act encoded as relevant became irrelevant in a context of retrieval. In this case the task could still be solved with the previous means, but it was sub-efficient within the changed constraints. An action or means is perceived as sub-efficient when the observer understands that with the specific means the given goal could be attained, however the observer is also aware that this solution is not the most efficient one [10]. Hence, adaptation could be achieved through an update of the previous means’ relevance (and consequently in the retrieval of the stored memory), within the modified constraints.

The other direction of change was that a previously irrelevant act or element of the observed behavior became relevant (and necessary) within new constraints. In this case recall and reevaluation of the previously irrelevant act was essential as the task could not be solved without it. Reliable and successful retrieval in such a case reflects a re-evaluation of the relevance of a step based on the causal-temporal structure of the original episode and thus arguably reflects the hallmark of episodic memory.

Results showed that in the short-term test 2-year-olds imitated selectively, and in the long-term retrieval test they were able to partially update their strategy to attain a goal by omitting a previously relevant action that has lost its relevance in the novel context. Critically, 2-year-olds were less flexible and could not always recall a previously irrelevant action, which gained relevance within the changed constraints. Based on the results it was supposed that imitative flexibility at the age of two is emerging but is still limited. Children readily updated when it simply required omitting a previously used action, but retrospectively recollecting and reconsidering an act caused difficulties for them. As an additional control condition of the study showed, recall of the irrelevant action decreased even immediately after the demonstration, indicating that at this age the causally irrelevant actions are omitted already at encoding [32].

This pattern of results was interpreted as 2-year-olds being able to recall specific past *events* that they experienced only once, showing the capacity of event memory, but not being able to rely on the identification of certain events *as* specific past events, that would help them initiate a guided search in their memory. This latter would require the ability of metarepresentation and an ‘act of remembering’, enabling episodic memory [33], and thus remembering the causal structures of the original episode, including the reasons why a specific action step was deemed irrelevant at the time. This raises the question of when children would show fully flexible updating of their actions by recalling and re-enacting a previously irrelevant action step. Such an update requires episodic recollection by recalling the original causal analysis of the scene during encoding that led to the omission of that step, re-evaluating the current action steps, and finally incorporating the previously omitted step now as an efficient (and necessary) means to attain a goal.

At what age might we find indications of such flexible memory use? There is some evidence indicating that at three years of age children can update their representations based on re-evaluating past events using episodic memory [34]. There, 3-year-old children were able to retrospectively revise the attributed belief of a partner wearing sunglasses when they themselves learnt that the sunglasses the partner had been wearing were opaque, and thus she could not have witnessed the ongoing events. In line with this, in Liszkai-Peres, Kampis and Király [32] some 2-year-olds were able to update their means, though this was not the dominant behavior at that age. Based on these studies, it should be expected that from around the fourth year of life, children may be able to flexibly update in a goal attainment situation with changed contextual constraints in delayed memory scenarios. The flexibility of memory is still a developing skill at the age of three, and based on episodic foresight studies it works only with shorter intervals [27]. However, episodic foresight includes planning as well which might put a further load on children [29]. Scenarios that focus only on past events and their consequences for the present might be a less demanding task as opposed to the episodic foresight tasks and thus may be more suitable for probing memory flexibility in children. Based on this, we could expect that 3-4-year-old children would be able to flexibly evaluate the relevance of action steps both at encoding and at delayed retrieval, and re-enact the necessary actions in a delayed imitation scenario.

At the same time, as children grow older, they tend to imitate not only the goal of an action, but also the exact means leading to it [15, 35]. This pattern is most apparent in the phenomenon of over-imitation, when children blindly copy all of the actions, even the completely irrelevant, arbitrary ones of a model (for a review see [36]). The tendency towards high fidelity imitation is governed by both cognitive and motivational processes, but mainly rooted in the development of normative action parsing and the growing motivation for affiliation [36]. The pedagogical setting of a situation, in which the model communicates with ostensive-referential cues, contributes to this effect [37]. In addition, from very early on, children rely on the demonstration of adults about the function of artifacts in order to narrow down their possible usage [38], and do not evaluate the causal function or necessity of an action step or element. Note, while in overimitation studies the imitated extra steps are completely irrelevant in relation to goal attainment, in the functional use of artifacts (and similarly in [32]), the irrelevant steps or solutions could be sub-efficient, yet lead to successful goal attainment. Still, like in overimitation studies, despite the means being obviously sub-efficient, older children stick to it, while younger ones are more likely to change their behavior [39]. Based on these, if the old means still works and thus there is no trigger to induce the reevaluation of previous actions, 3-4-year-old children might stick to a former solution they observed in a communicative scenario, instead of focusing on the efficiency of the means within the new context. Therefore 3-4-year-old children might be less selective than 2-year-olds especially in the second session, when a prior relevant act that was demonstrated to them ostensively becomes irrelevant.

In the present study we investigated whether 3-4-year-olds can accommodate their behavior to the changing situational requirements after a 1 week delay. In the Irrelevant-Relevant condition in Session 1 the tool use was irrelevant, and it gained relevance in Session 2. In the Relevant-Irrelevant condition the constraints were reversed: in Session 1 the tool use was relevant, and in Session 2 it lost its relevance.

A control condition was also introduced to test the effect of short-term context change (Short-term change to Relevant condition) to investigate the effect of context change shortly after demonstration (already in Session 1) and thereby eliminating possible effects of long-term delay. Finally, a baseline condition was designed to measure frequency of tool use without demonstration in the relevant constraint (Relevant Baseline).

Furthermore, to investigate the developmental trajectory of memory flexibility we also performed a comparative analysis based on the data from the study with 2-year-olds by Liszkai-Peres et al. [32], and the data of the present study with 3-4-year-olds.

## Method

The experimental design, materials and procedure are based on a previous study with 2-year-olds [32].

### Participants

The participants of this study were N = 66 white, monolingual preschoolers living in a large capital city in Europe. Children’s ages ranged from 33 to 54 months, with a mean of 43.5 months (*SD* = 4.1 month). A further 18 children were tested but excluded from all analyses due to passivity (6), fussiness (3), experimenter error (4), or participation only in one test instead of two in conditions requiring two test occasions (5). In the final sample, 18 children (8 boys) were in the Irrelevant-Relevant condition, 18 children (12 boys) in the Relevant-Irrelevant condition, 12 children (4 boys) in the Relevant Baseline condition, and 18 children (8 boys) in the Short-term Context Change to Relevant condition.

The study was approved by the Research Ethics Committee of the University. The parents of all participating children gave written informed consent before participation. Children were given a small gift for their participation. Although the study did not intend to investigate cultural or demographic effects, low variability is a limitation of the study.

### Materials

**Fig 1:**
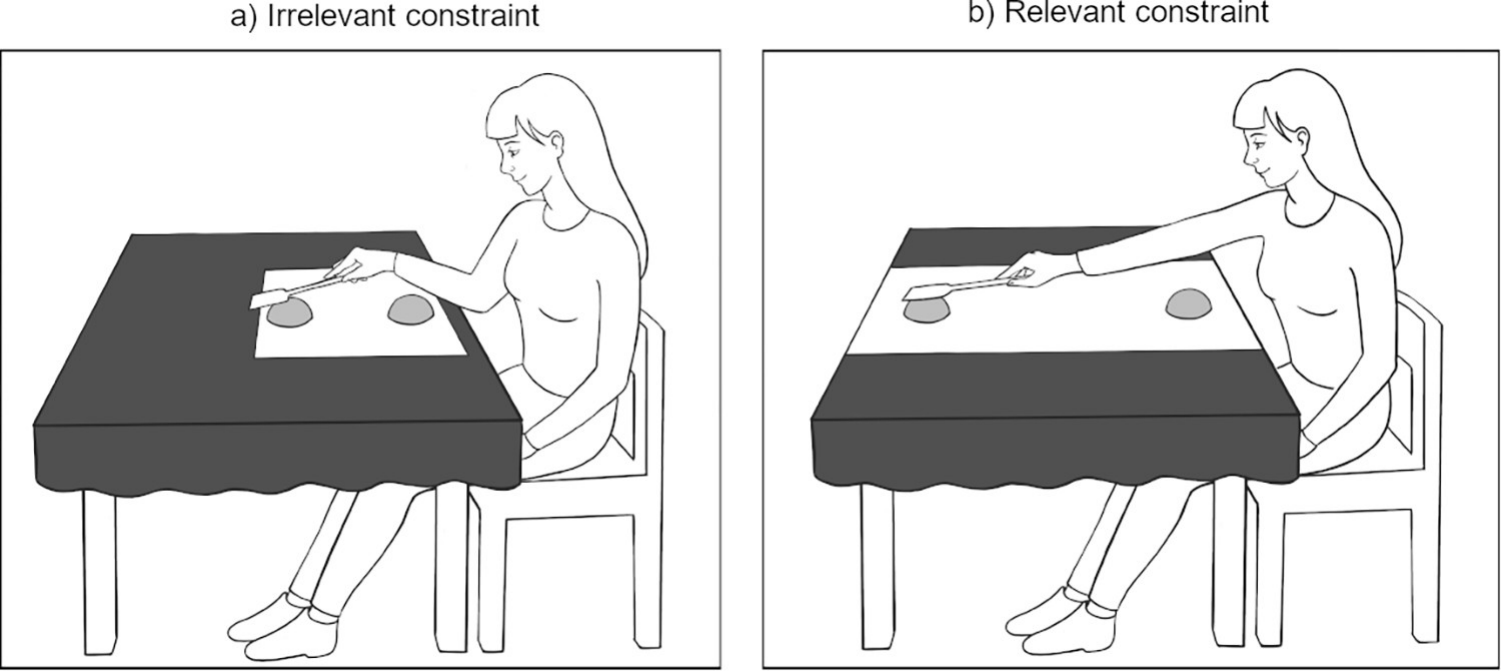
Irrelevant and relevant constraints during demonstration phase. One part of the objects was always close to the experimenter/participant; the goal was to collect the other object part. In the irrelevant context the other part of each pair was within arm’s reach, thus the situational constraints made the tool use unnecessary. In the relevant context the second part was further away and could not be reached by hand, thus tool use was necessary.

The object set contained three toys with removable parts and a white wooden spoon (see Fig 1 in S1 File). Each toy had two separable parts, neither of which could fulfill the toy’s function alone. There was a plastic propeller with a green head part (20 cm long) that could be detached from an orange stick (19 cm long), and after joining the parts, the propeller could be whirled. Another object was an orange polystyrene ball (4.5 cm diameter) cut into half, with hidden magnets. Once the halves stuck together, the ball could be thrown by one hand and caught by the other like a regular ball. The third object was a bicycle horn (16 cm long) consisting of a plastic tube (9 cm long) and a rubber end (7 cm long). After assembling the horn, the rubber end could be squeezed to make a loud sound.

Demonstration and tests were completed on a table (76 x 106 x 52 cm). The separated parts of the toys were placed in a detached form onto one of two white rectangular panels (long panel 74.5 x 29 cm; short panel 28 x 29 cm) on a black tablecloth (145 x 200 cm). One part of the toy was always as close to the participants as possible, the other part was always further away, either within arm’s reach for the child (ca. 30 cm) (irrelevant constraints for tool use) or out of reach (ca. 70 cm) (relevant constraints for tool use). Distance was modified according to the abilities of the children (e.g. for a tall, long-handed child the object was placed further away). A wooden spoon (30.5 cm long) was placed on the right side of the table as an aid for reaching target objects (Fig 1).

### Design and procedure

**Fig 2:**
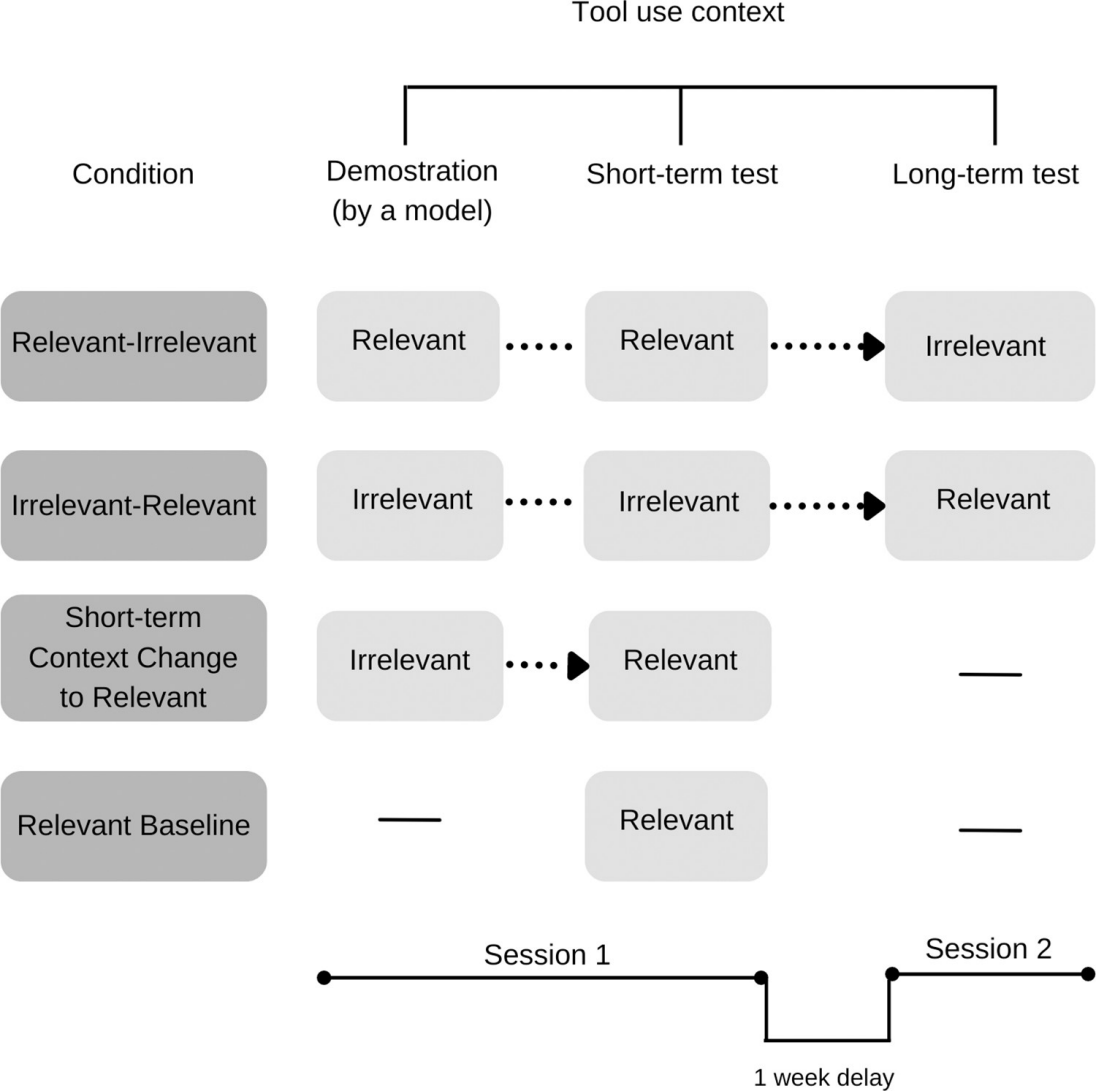
Experimental design of the study. Conditions could consist of three phases: demonstration, short-term re-enactment, and delayed re-enactment. The demonstration and short-term re-enactment took place at the same occasion, in Session 1. One week later, in Session 2 only the delayed re-enactment took place. Tool use context (relevant/irrelevant) changed between or within sessions according to the conditions.

The study included three experimental conditions and a baseline condition. Each experimental condition started with a demonstration, which was followed by an imitation test after a short-term delay. Two experimental conditions also included an additional deferred imitation test with a changed context, which was carried out one week after the first session (for a detailed description of the design see Fig 2). The third experimental condition only involved the short-term re-enactment in a changed context. In the baseline condition, there was no demonstration of tool use (see below). Children were randomly assigned to a condition. The experiments were conducted by one of three experimenters (two women and a man).

### Irrelevant-relevant condition

In the initial state children sat on the caregiver’s lap, next to the experimenter. The props were put on the table out of reach for the child, and parents were also asked to prevent children from touching them during demonstration. The experimenter sat in front of the white panel of the table on which each of the toy sets were placed earlier, their parts separated.

In the demonstration (first part of Session 1), the experimenter pointed to the part of the set at the closer end of the table saying, “Look, this is one part of the toy!”, then pointed at the other part of the set placed further saying, “Look, there is the other part of it!”. Then, she grabbed the wooden spoon, showed it to the child, and reached the target object with it. Importantly, the other part was close to her (within arm’s reach). After reaching the part, she assembled the two parts, showed the function of the toy (e.g., the propeller whirling, throwing and catching the ball, the horn making a sound) and then put it away under the table. She demonstrated the assembly of the remaining two objects with the same procedure. The toys were presented in two predefined orders (order A: propeller, ball, horn; order B: horn, propeller, ball), and the part children needed to retrieve was the same for each child: the head of the propeller, the rubber end of the horn, and one part of the ball (the ball’s two parts were identical).

A short-term delay test followed the demonstration (second part of Session 1). At the beginning of the test the experimenter and the participant switched places. Then only one of the object sets was placed (in detached form) onto the cleared table in the same position as it was during the demonstration (within hand’s reach for the child, as well), and the child was encouraged to play with it (“Now, you can play with it!”). If the child did not initiate any action, the experimenter tried to call the child’s attention to the object part by pointing and saying, “Look, there is the other part of it!”. After assembling one toy, the experimenter hid it under the table and put another object set onto the table. Children received the toys in the same order as they were demonstrated (order A or B, see above).

Session 2 was conducted one week later and the child had to re-enact, without prior demonstration, the actions presented one week earlier. The task remained the same as before: to retrieve the second part and assemble the objects. The difference between the two sessions was that the target objects get further away than earlier, changing the context relevant for tool use.

### Relevant-irrelevant condition

The procedure was nearly the same as the previous Irrelevant-Relevant condition, but here the relevance of tool use was reversed between sessions. This means that tool use was relevant during the demonstration and the short-term reenactment test (Session 1), and became irrelevant one week later, in the delayed imitation test (Session 2).

During demonstration (S1) one part of a toy was out of arm’s reach for the model (relevant context for tool use). To emphasize the distance of the target object, the model first tried to reach it by stretching her hand as far as she could. After recognizing her unsuccessful action (“Uh-oh”), she grabbed the wooden spoon, got hold of the target object with it, showed the function of the toy, and then repeated the demonstration with another toy (toys, functions, demo order of the toys were the same as in the Irrelevant-Relevant condition).

During the short-term imitation task (S1) the object was also far for the child making the tool necessary to use for goal attainment (relevant tool use context). If participants tried to attain the distant part only with their hands, after 1 minute (or if the child lost interest in obtaining the object) the experimenter took the current set away and placed the next set on the table (this happened in 3.5% of the trials in the relevant context (7 out of the 198 relevant context trials)) (see also Table 1 in S1 File for details on success in tool use). If children used the tool in a subsequent trial, the experimenter placed the previously unsuccessful trial’s object set in the original distant position (1.5%– 5 out of the 198 relevant context trials). Successful tool use in these additional trials were counted into the total number of tool use trials for that child. If children did not use the tool in any of the three trials, after the third trial both of the object parts for each set were placed within arm’s reach of the child, to assess whether children would assemble the sets (1%– 2 out of the 198 relevant context trials).

One week later, In Session 2, no demonstration was displayed, and the other part of the target object got closer, within hand’s reach making tool use irrelevant or at least a sub-efficient means for goal attainment.

### Short-term Context Change to Relevant condition

In the Short-term Context Change to Relevant condition only one session was conducted. Here, demonstration was performed in an irrelevant context for tool use (objects were close to the model), but in the short-term imitation task the constraints were modified so as to make tool use relevant for tool use (objects got out of arm’s reach). Here, the least effortful means for reaching an object, hand use did not work.

### Relevant Baseline

The Relevant Baseline only included one session. The test was carried out with the same object set and tool, but there was no demonstration of reaching with the tool or demonstration of assembling the objects. However, the experimenter indicated that the two parts belong together by showing the object’s function in attached form (e.g., whirling, throwing, making sound) before taking it apart and putting the parts onto the table. To make the use of the object relevant, the target part was out of the children’s reach. The instruction was, “Let’s play with them!”. If children were passive for a long time, the experimenter pointed to the other piece saying, “This is the other part of the toy, let’s play with it!”, to motivate the child to retrieve the object.

### Coding

**Fig 3:**
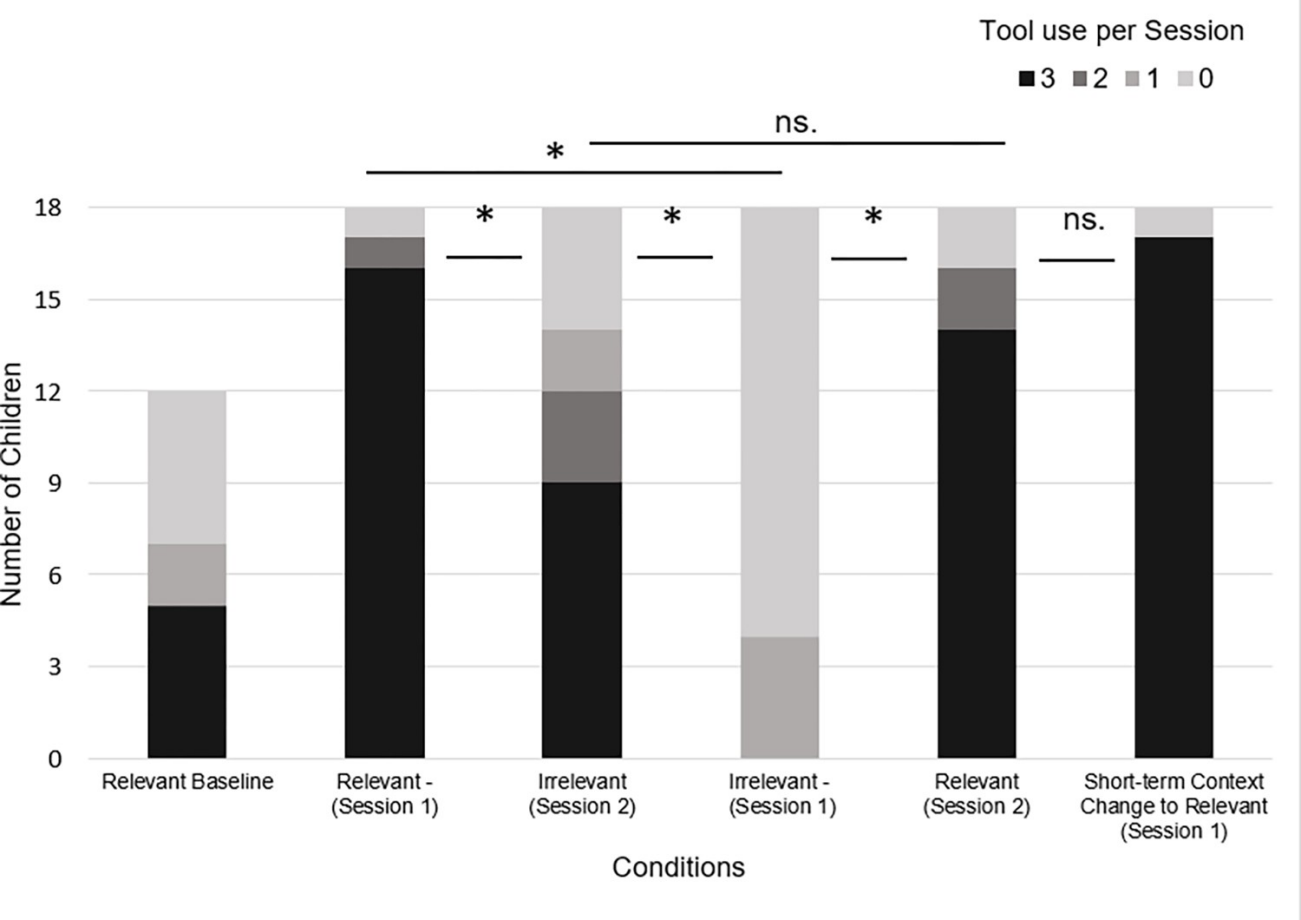
Tool use in experimental and control conditions. Tool use score varied from 0 to 3. The figure includes tool use scores of Session 1 and Session 2, in the Relevant Baseline, Relevant-Irrelevant, Irrelevant-Relevant, and Irrelevant-Relevant Short-term conditions. Preschoolers either used a tool or reached for the object by hand. Asterisks indicate a significant difference * *p* < .05.

Test videos were coded off-line. For each trial, children were given a score of 1 if they used the tool (target act), thus the total number of tool uses (dependent variable) ranged from 0 to 3. A behavior was coded as “tool use” if the participant grabbed the tool and reached with it toward the second object part regardless of whether this movement was successful or not (see also Table 1 in S1 File on the success of tool use). In 98% of the trials coded as tool use, preschoolers were able to attain the object with the tool and in only 2% of the trials were their attempts to reach the object with the tool unsuccessful. Only those participants who reached toward the object part either by hand or with the tool were included in the dataset. As such, behavior was coded as "lack of tool use" if children used their hands for obtaining the object. Children were excluded from the dataset if they did not try to obtain the toys (“passive”), did not pay attention during demonstration, refused to play altogether, or quit the test session before completing it (“fussy”) (see Participants section). Joining object parts after obtaining both pieces was not an inclusion criterion, but children successfully assembled the object sets in 99% of the trials, and in 76% even without a prompt (see details in Figs 2, 3 in S1 File). Generally, assembling the toys was easy for children, given that this procedure was unsuccessful in only 1% of all the trials. The children either requested help (2 trials out of a total of 5 unsuccessful trials), lost interest (2 trials out of a total of 5 unsuccessful trials) or did not know what to do (1 trial out of a total of 5 unsuccessful trials).

Inter-coder reliability was high in the Irrelevant-Relevant and Relevant-Irrelevant conditions (Cohen’s kappa = .94, *p* < .001). Another independent coder coded 50% of the Irrelevant-Relevant Short-term videos and 50% of the Baseline videos. Reliability between coders was good (Cohen’s kappa = .93, *p* < .001).

## Results

SPSS 25.0 for Windows was used for statistical analysis, and *p* < .05 was accepted as significant throughout. The dependent variable was whether children performed the target action (tool use). For the Generalized Linear Mixed Model (GLMM) analyses we included trials as a random factor, therefore the analyses were executed on the scores children received for each trial (0 or 1), while in all other analyses the sum of trial scores (0–3) was used.

As the design of the current study was based on a former study [32], and data of 2-year-olds was available online we compared directly the results of the two studies at the end of the Results section. While exploratory, we hope that the result gives important insights about transitional changes in memory flexibility and in imitation, and also motivates further studies in the future.

GLMM with binary regression was used to test whether tool use can be explained by Condition (Irrelevant-Relevant, Relevant-Irrelevant, Short-term Context Change to Relevant, Relevant Baseline), Session (1, 2), Trial (1, 2, 3), Sex (boy, girl), Age, Object order (A, B) and Experimenter (Experimenter 1, 2, 3). The initial model included main effects and the interaction of Condition and Session. We used backward elimination for model selection. Then, we used planned comparisons to explain the model further.

The GLMM showed Condition x Session interaction *F*(1, 300) = 51.28, *p* < .001, Cohen’s *f* = 0.41, whereby children used the tool differently between sessions depending on the condition (which differed by constraint change).

### Comparison of tool use before and after context change

To assess the changes in tool use between sessions, we compared Session 1 and Session 2 in the two experimental conditions separately, with a Wilcoxon Signed Rank test. In the Irrelevant-Relevant condition children used the tool in Session 2 (*Mean* = 2.72, *SD* = 0.75) significantly more frequently than in Session 1 (*Mean* = 0.17, *SD* = 0.38), *Z* = -3.82, *p* < .01, *r* = -0.63. In contrast, in the Relevant-Irrelevant condition children used the tool significantly less often in Session 2 (*Mean* = 1.94, *SD* = 1.26) compared to Session 1 (*Mean* = 2.78, *SD* = 0.73), *Z* = -2.57, *p* = .01, *r* = -0.43 (Fig 3). In light of these results, children reacted to the constraint changes in both conditions: the frequency of their tool use differed significantly between Session 1 and 2.

### Tool use in the short-term tests

Next, we probed whether within the two sessions children’s propensity for tool use differed depending on the condition (and thus as a function of the tool’s relevance). With a Kruskal-Wallis test we compared conditions within Session 1 and 2 separately. In the analysis of Session 1 we used tool use as the dependent variable and condition as a between-subjects variable (Irrelevant-Relevant S1, Relevant-Irrelevant S1, Short-term Context Change to Relevant, Relevant Baseline S1). In Session 1, conditions differed significantly in tool use, *H*(3) = 43.07, *p* < .001, η^2^ = .65. Dunn’s pairwise tests with Bonferroni correction revealed that Relevant Baseline (*Mean* = 1.17, *SD* = 1.4) differed from the conditions where tool use was relevant (Relevant-Irrelevant (*Mean* = 2.78, *SD* = 0.73), *p* = .019; Short-term Context Change to Relevant (*Mean* = 2.83, *SD* = 0.71), *p* = .011), but not from the condition where the tool use was irrelevant in this first session (Irrelevant-Relevant (*Mean* = 0.17, *SD* = 0.38), *p* = .346).

Moreover, in Session 1 the condition in which tool use was irrelevant (Irrelevant-Relevant, *Mean* = 0.17, *SD* = 0.38) differed from the conditions in which it was relevant (compared to Relevant-Irrelevant (*Mean* = 2.78, *SD* = 0.73): *p* < .001; Short-term Context Change to Relevant (*Mean* = 2.83, *SD* = 0.71): *p* < .001) (Fig 3). Thus, in Session 1 children used a tool significantly more in the conditions where it was relevant for goal attainment and was demonstrated before, than if the tool use was not relevant or had not been demonstrated. The difference between the Irrelevant-Relevant and the Short-term Context Change to Relevant condition indicate that children at the age of 3 and 4 take into consideration the situational demands during imitation, as after the irrelevant demonstration they used the tool more, if it became relevant (Short-term Context Change to Relevant) compared to the situation where it remained irrelevant in the short-term test (Irrelevant-Relevant condition).

### Tool use in the long-term tests

For analyzing Session 2, we compared conditions with a Kruskal-Wallis test with tool use as the dependent variable and condition as a between-subjects variable (Irrelevant-Relevant S2, Relevant-Irrelevant S2, Relevant Baseline S1), and likewise it showed that conditions differed in tool use, *H*(2) = 9.95, *p* = .007, η^2^ = .18. Post-hoc analyses with Bonferroni correction indicated that Relevant Baseline (*Mean* = 1.17, *SD* = 1.4) differed from the condition in which tool use was relevant in Session 2 (Irrelevant-Relevant (*Mean* = 2.72, *SD* = 0.75), *p* = .012), but not from the condition in which it was irrelevant (Relevant-Irrelevant (*Mean* = 1.94, *SD* = 1.26), *p* = 1). Comparing the two experimental conditions, the second session of the Relevant-Irrelevant (*Mean* = 1.94, *SD* = 1.26) and the Irrelevant-Relevant (*Mean* = 2.72, *SD* = 0.75) conditions did not differ significantly from each other, *p* = .321 (Fig 3). These results suggest that in Session 2, children used the tool more often if it was demonstrated to them before in the condition where the tool use became relevant, compared to when it was not demonstrated. However, while they were able to retrieve the tool use from memory when the constraint change proved it to be relevant, children still used the tool in the situation when it lost its relevance with comparable frequency. To confirm this claim we compared tool use in the irrelevant context of the two experimental conditions (Session 1 of Irrelevant-Relevant and Session 2 of Relevant-Irrelevant). A Mann-Whitney U test showed difference *U* = 45, *p* < .001, η^2^ = 0.47, children used the tool more often in the irrelevant context during Session 2 (*Mean* = 1.94, *SD* = 1.26) than during Session 1 (*Mean* = 0.17, *SD* = 0.38).

### Tool use in relevant and irrelevant context

With this analysis we compared the relevant contexts of Irrelevant-Relevant and Relevant-Irrelevant conditions, and also the irrelevant contexts of these two conditions. In the relevant context, results did not show any difference between the two relevant situations (Mann-Whitney U = 153.5, p = 0.791, n2 = 0.002). In the irrelevant context we found that the two conditions differed (Mann-Whitney U = 45, p < 0.001, n2 = 0.38), children used the tool more if it was preceded by a relevant session (Relevant-Irrelevant Mean = 1.94, SD = 1.26) compared to the condition when it was not (Irrelevant-Relevant, Mean = 0.17, SD = 0.38).

### Comparison of tool use after short-term and long-term context change

Finally, with a Mann-Whitney test we analyzed whether there is a difference between constraint changes that happen after a long-term or short-term delay after demonstration in the irrelevant tool use context. Comparison of Irrelevant-Relevant (*Mean* = 2.72, *SD* = 0.75) and Short-term Context Change to Relevant (*Mean* = 2.83, *SD* = 0.71) conditions showed that timing of constraint change did not influence tool use imitation, *U* = 145, *p* = .324, η^2^ = .01 (Fig 3).

### Comparison of 2—and 3-4-year-old children’s performance in a short-term and long-term delayed imitation task

We executed further analyses to directly compare the performance of 2-year-olds and 3-4-year-olds (both datasets are available online, previous dataset with 2-year-olds [32] and the current dataset with 3-4-year-olds). We involved only the two experimental conditions (Relevant-Irrelevant, Irrelevant-Relevant) from both studies (2-year-olds: *N* = 36 (18/condition), *Mean age*: 24.33 months; *SD* = 1.23 month; range: 22–27 months; 3.5-year-olds: *N* = 36 (18/condition), *Mean age*: 43.56 months; *SD* = 4.61; range: 33–54). GLMM was applied with Tool use as a dependent variable, and Age (continuous variable), Condition, Session as independent variables. Results show Age x Session interaction (*F*(1,424) = 5.92, *p* = .015, Cohen’s *f* = 0.11) indicating that performance differed by age in the two sessions of the studies. Moreover, a Condition x Session interaction (F(1,424) = 5.5, p = .019, Cohen’s f = 0.1) was found.

Based on the latter result we also compared the performance of the two samples in the two conditions separately. In the Irrelevant-Relevant condition, GLMM with tool use as a dependent variable and Session (first or second) and Age group (2 or 3-4-year-olds) as independent variables showed a significant interaction of Age group x Session *F*(1, 212) = 6.49, *p* = .012, Cohen’s *f* = 0.16. Post-hoc analysis showed a difference in Session 2 between age groups *t*(212) = 2.39, *p* = .017, meaning that older children (Mean = 0.85, SD = 0.36) used the tool more in the delayed test—if the new situation required it—compared to younger ones (Mean = 0.54, SD = 0.5) (see Fig 4 in S1 File).

In the Relevant-Irrelevant condition, applying the same GLMM analysis we found the main effect of Session (*F*(1,214) = 38.32, *p* < .01, Cohen’s *f* = 0.42). This result indicates that the two age groups similarly used the tool more in the relevant Session 1 (2-year-olds *Mean* = 1, *SD* = 0; 3-4-year-olds *Mean* = 0.93, *SD* = 0.26) and less in the irrelevant Session 2 (2-year-olds *Mean* = 0.35, *SD* = 0.48; 3-4-year-olds *Mean* = 0.65, SD = 0.48) (see Fig 5 in S1 File).

We conducted a confirmatory analysis for these results with a more stringent criterion: the original tool use score (0–3) was recorded into a binary score 0/1 (1 if the child used the tool in all three trials, in any other cases 0). Results of this analysis can be found in the Section 3 in S1 File.

## Discussion

In the present study we probed 3-4-year-old children’s use of episodic memory in a delayed imitation paradigm. Between the demonstration and short-term re-enactment in Session 1, and the delayed re-enactment in Session 2 (a week later), we introduced a change in the scenario such that the relevance of an action step, the use of a tool, changed. Our results showed overall that children imitated the goal-directed action by considering the constraints of the context. We propose that they re-evaluated the necessity of an action step based on the constraints in a previous event, indicating flexible episodic memory capacities at this age.

In Session 1 children observed the use of a tool in a relevant or irrelevant context. Here, the imitative strategy of the two experimental groups differed clearly in accordance with the situational constraints: if the tool was relevant for goal attainment, they used it, but when it was unnecessary, they omitted the tool use. This finding is similar to the pattern found with 2-year-olds [32], and in line with studies demonstrating the importance of efficiency evaluation in goal attainment during imitation [8–10]. In accordance with the present finding, in the study of Fong et al. [40] 4- to 6-year-old preschoolers chose the more efficient tool in a goal-attainment situation, even though the other less efficient but functionally working tool was supported by normative labels (e.g. “everybody uses this”). Based on these results, we provide further evidence that in the case of tools, instrumental and efficiency cues could have a stronger influence on behavior than social or normative cues [41]. It is also possible that in the applied paradigm tool use was not as arbitrary as actions in typical overimitation tasks (see the introduction part about sub-efficiency) and thus more readily evaluated with regard to its necessity or efficiency. Overall, these results contribute to the larger picture of imitation being context dependent [36].

The main finding of the present study is the highly flexible behavior and reliable retrieval of a previously irrelevant action step based on the re-analysis of the causal structure of events in 3-4-year-old children as indicated by the fact that they were able to retrieve a formerly irrelevant act at nearly ceiling level both after a short (several minutes) or long (one week) delay. When the constraint changed from irrelevant to relevant shortly after demonstration (Short-term Context Change to Relevant), children in the present study used the tool almost at a ceiling rate. This result further supports the claim that following a shorter interval, preschoolers show high behavioral flexibility in order to accommodate a situation for successful goal attainment [24, 26, 34]. At the same time the function of the tool was more or less straightforward and children may have used it easily for goal-attainment. Thus it would be interesting to include more arbitrary or unusual actions in the future to probe the degree of children’s flexibility.

In the long-term deferred imitation conditions, where one week passed between the first and second sessions, the behavior of the preschoolers also adapted to the modified context, as indicated by the significant difference in rate of tool use between the first and second sessions in both conditions. When this action step became relevant a week later (Relevant-Irrelevant condition), while 2-year-olds [32] already showed flexibility albeit in a limited way, children in the present study showed flexible and reliable retrieval of the critical action step at the age of 3 and 4. This result provides evidence that 3-4-year-children’s memory capacities enable them to successfully and reliably re-evaluate previously discarded action steps and incorporate them into their actions in a changed context. What may explain the difference in behavior between age groups? On one hand, it could be the result of the quantitative development of event memory, as memory capacity is increasing with time [6, 7]. However, besides event memory capacity growing, other cognitive skills also likely contribute to the flexible behavior of preschoolers. As Liszkai-Peres, Kampis, & Király [32] argue, relying on event memory may enable remembering elements of original events, but this may be somewhat arbitrary or unreliable. According to Mahr & Csibra [33] at the age of 3–5 children begin to understand that events are the source of their beliefs. It may be the emergence of this capacity, termed the remembering attitude—potentially together with the gradual development of event memory—that allows children to realize that in the novel context the solution could only be found via retrieving the specifics of the original situation, that could be classified as episodic memory, leading to a reliable and flexible performance at this age.

These results are in line with Király et al. [34], where 3-year-old children were able to revise their previous beliefs, if they got acquainted with new information influencing the interpretation of a previous situation. The results also strengthen the findings of Williamson & Meltzoff [24], where children could easily apply an irrelevant step presented by a model that became necessary in the test phase shortly after the demonstration. Moreover, the delay introduced in our study expands this kind of flexibility from short-term to long-term memory processes, as children were able to access former knowledge even after a week delay. In addition, flexibly manipulating elements of an event seems to be an easier task for 3-4-years-olds when done retrospectively as opposed to doing so in episodic foresight tasks [27, 29]. This retrospective manipulation might be a clearer index of episodic memory capacity as this task does not require the use of executive functions like planning as much.

Children in the present study tended to show more flexibility in their strategy change in the Irrelevant-Relevant condition–where children were able to recall the previously irrelevant means–and showed slightly more inflexible behavior in the Relevant-Irrelevant condition–where they were less prone to skip the unnecessary tool use. Using a stricter criterion, looking at whether children used the tool in all three trials showed that 3-4-year-olds used the tool more compared to 2-year-olds (see S1 File for this additional analysis). Indeed, in the 3-4-year-olds, despite the difference in tool use between Session 1 and Session 2 in both experimental conditions (Relevant-Irrelevant, Irrelevant-Relevant), rate of tool use in the second sessions did not show any difference. This lack of difference stems from the fact that in the delayed reenactment phase children in the irrelevant situation often continued the tool use even though it was sub-efficient, while in the condition where S2 involved the relevant context children changed their previous strategy and started to use the tool.

Why did the 3-4-year-olds not omit the tool use more in the delayed reenactment phase when it became irrelevant? A possible explanation is that they encoded the relevant tool use in the first session more normatively, in line with previous research indicating that children in this age tend to follow norms more than younger ones [36, 38, 42–45]. Such normativity may manifest in the form that children in the current study would be more likely to retain an action step in memory that they have previously seen relevant. As in the second session of the Relevant-Irrelevant condition the constraint change in this condition does not motivate strategy change as much as in the Irrelevant-Relevant context, where the previous solution of the children did not work anymore, this may contribute to normativity manifesting in this condition. Specifically, in the Relevant-Irrelevant condition the constraint change in Session 2 continued to allow the use of the previously working means from Session 1, only less efficiently. Preserving the already learned solution could indicate that in the long-term children associated the tool as a functionally and socially accepted way of goal attainment taught by the model, and thus they tended not to question its relevance anymore unless they had to [46].

From preschool years onwards humans tend to stick to former successful solutions and often do not question the reasons behind an act. This is illustrated in the anecdote of Sylvia’s recipe, where the protagonist had always cut the end of the ham before putting it into the oven, just like she learned from her mother, only to find out later from the mother that she only did so because she did not have a large enough pan [44]. In this story, Sylvia re-enacted a successful observed strategy without evaluating or questioning whether each step is necessary for the successful outcome (in the story, the delicious ham). In line with this, in the study of Carr, Kendal, and Flynn [42] efficacy of a goal reaching behavior was varied. They found that between the age of 4 to 9 years children copied even the least efficient behavior instead of innovating a new means [42]. Moreover, learning about a tool is often part of cultural knowledge; its logic sometimes appears to be cognitively opaque, so high fidelity imitation is an adequate strategy and a quick solution for learning about such cultural artifacts [38, 43, 44]. Social factors like norms also strengthen the preservation of a previous means [47], and could result in over-imitation [36]. While we did not observe over-imitation in the first session, as indicated by the difference in rate of tool use between irrelevant and relevant contexts in Session 1, it is plausible that once children re-enacted tool use as a successful strategy obtained in a communicative context, they stored it as part of the action sequence and did not re-evaluate its necessity in Session 2. These results highlight the interaction between children’s memory capacities and the situational factors that trigger them: children in our study clearly seemed capable of flexibly re-evaluating the necessity of action steps and adjusting their behavior, but their tendency to apply these skills was modulated by the context.

## Conclusions

Deferred imitation was the first method to explore the mnemonic capacity of nonverbal children[21, 22], and it is a behavioral indicator of adult memory as well [48]. It is generally accepted that deferred imitation taps into declarative memory, the system responsible for storing knowledge and events that could be retrieved consciously later [21]. However, it is often debated whether deferred imitation could be an indicator of episodic memory, which is responsible for preserving one-time experiences in a detailed manner and allows re-experiencing earlier events [49]. We have argued that with the flexibility task presented here we are more likely to tap on episodic memory, as preservation of a long-term memory, including irrelevant elements, and especially the flexible reevaluation process likely reflects the attitude of remembering and thus the operation of an episodic memory [33]. Moreover, our findings point to the direction that memory processes behind imitation might work on an inferential basis as the goal attainment event from a week earlier clearly interacted with the current situational constraints and efficacy cues. Flexible information access as part of episodic memory is supported by the inference-based account of Klein et al. [50]. They propose that a possible function of episodic memory is this kind of reevaluation process: the refinement of inferences originating from past experiences in order to make better predictions for the future.

In sum, in the current study 3-4-year-old children’s memory showed some signature features of episodic memory, as they were flexible enough to retrieve and revise an irrelevant act, if it was necessary in a novel context. At the same time, they seemed to rely greatly on former experiences from a communicative demonstration, and if the context allowed for their previous solution to be used, they were less likely to deviate from it. In an earlier study, 2-year-olds were not able to fully incorporate a previously omitted action into their behavior. Together, these findings indicate that the behavior of children in the preschool years is increasingly facilitated by their developing memory capacities, and influenced by the context they are applied in.

## Supporting information

S1 File (DOCX)
