# Supplementary material for: 3-4-year-old children’s memory flexibility allows adaptation to an altered context
Source: PLoS One. 2022 Sep 23;17(9):e0275071. doi: 10.1371/journal.pone.0275071 (PMC9506616; doi:10.1371/journal.pone.0275071)
Supplement: S1 File — (DOCX) [file pone.0275071.s001.docx]

**Supporting information (SI Appendix)**

**3-4-year-old children’s memory flexibility allows adaptation to an altered context**


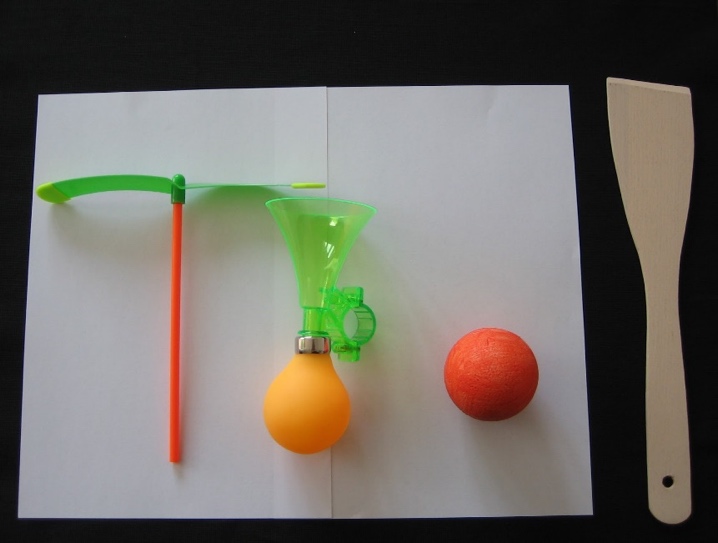


**Fig 1. Object sets**

Propeller (left), bicycle horn (middle), ball (right) on the white paper, and a wooden spoon for retrieval of objects (on the right).

1. **Efficiency of tool use**

Across experiments the operational criteria for tool use was reaching with the tool in the direction of the distant object. In the study in 201 trials children used the tool, most of the time successfully (196 out of 201 cases) and only in 5 out of 201 trials could infants not reach the object with the tool (e.g. due to manual problems), although they clearly tried to do it (for the number of successes on individual trials see Table 1).

**Table 1. Efficiency of tool use**

| **Condition** | **Tool use** | **Session 1**  (**actual tool use**/all trials) | **Session 2**  (**actual tool use**/all trials) |
| --- | --- | --- | --- |
| *Irrelevant-Relevant* | unsuccessful | **0**/54 | **5**/54 |
|  | successful | **3**/54 | **44**/54 |
|  | no use | **51**/54 | **5**/54 |
| *Relevant-Irrelevant* | unsuccessful | **0**/54 | **0**/54 |
|  | successful | **50**/54 | **34**/54 |
|  | no use | **4**/54 | **20**/54 |
| *Short-term Context Change to Relevant* | unsuccessful | **0**/54 | ̶ |
|  | successful | **51**/54 | ̶ |
|  | no use | **3**/54 | ̶ |
| *Relevant Baseline* | unsuccessful | **0**/36 | ̶ |
|  | successful | **14**/36 | ̶ |
|  | no use | **22**/36 | ̶ |

The table shows how many children used the tool and out of them who succeeded (successful tool use) or failed (unsuccessful tool use) to obtain object part B in the sessions of each condition. The number on the left shows the actual tool use, the number on the right shows the number of trials in a session (e.g. in the Irrelevant-Relevant condition 3 trials per session for 18 participants amounts to 54 trials in total; in baseline: 3 trials per session for 12 participants amounts to 36 trials in total).

1. **Assembling objects after obtaining the distant part**
   1. **Analysis of trials**

The overall goal of the demonstrated action was to put together objects with salient functions from two parts. To attain this goal, children needed to get the distant part of the object, which was within hands’ reach or out of hands’ reach (depending on the experimental condition and the constraint change). Though the experiments focused on the (re-)enactment of tool use, we also analyzed whether children successfully assembled the objects after obtaining the distant parts. In addition, it was coded whether children required a prompt to assemble the object parts.

In 99% of the trials (302 out of 306 trials) object parts were assembled after retrieval of the distant part. If children did not assemble the toy, they were encouraged to do so, but this process was needed in only 24% of the trials (72/306). (for a summary of object building and prompt to do so per condition, see Fig 2 and 3).

Altogether, these results strengthen the assumption that children understood the aim of putting together the objects and behaved accordingly. The procedure of assembling the parts did not seem to cause difficulties in general. From all trials in only 1% of the trials (4 out of 306 trials) did the children fail this task e.g. due to lack of interest (3), did not know what to do (1).


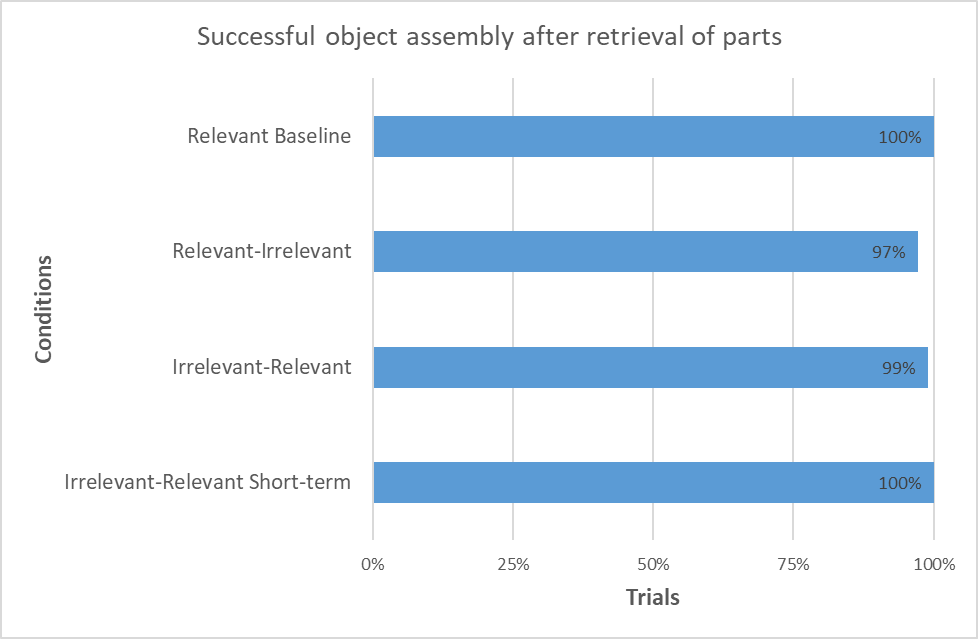


**Fig 2*.* Successful object assembly after retrieval of parts**. The figure shows the percentage of trials where object assembly was successful after retrieval of the distant part.


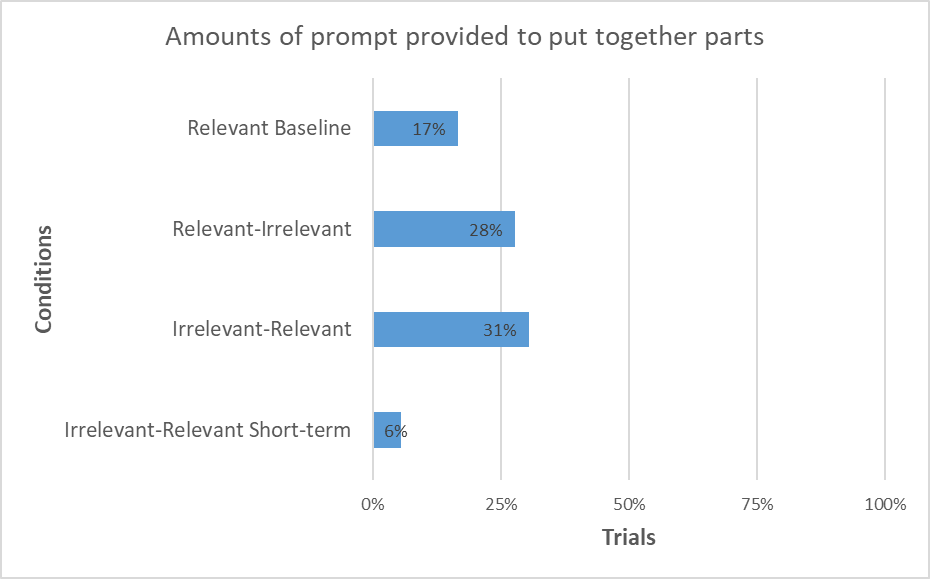


**Fig 3. Amounts of prompt provided to put together parts**. The figure shows the percentage of trials where a prompt was provided to assemble the objects after retrieval of the parts.

1. **Comparison of 2 - and 3-4-year-old children’s performance**


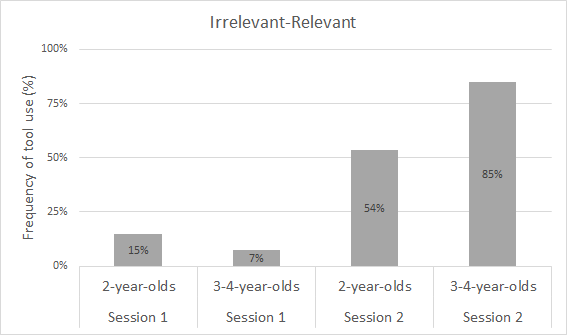


**Fig 4***.* **Frequency of tool use (percentage of tool use/all trials) of the two age groups in Session 1 and Session 2 in the Irrelevant-Relevant condition**


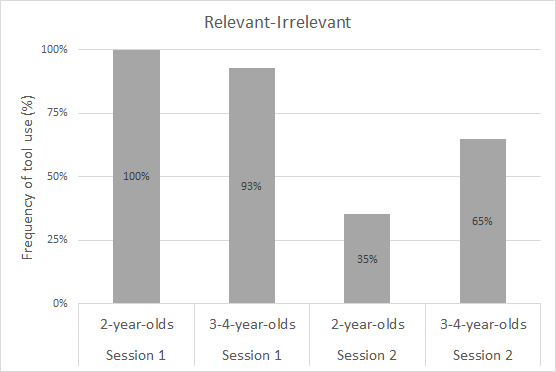


**Fig 5*.* Frequency of tool use (percentage of tool use/all trials) of the two age groups in Session 1 and Session 2 in the Relevant-Irrelevant condition**

As part of the motivation of the study was to probe the consistency of children’s behavior (a 3-trial-procedure was applied), the most stringent criterion is to analyze the number of children who used the tool in all three trials. In accordance with this the original tool use score (0-3) was recorded into a binary score 0/1 (1 if the child used the tool in all three trials, in any other cases 0). In the Relevant-Irrelevant condition, in Session 1 children showed similar frequency in tool use (Mann-Whitney *U* = 144, *p* = .584, *d* = 0.19), but performance in Session 2 was significantly different (Mann-Whitney *U* = 90, *p* = .022, *d* = 0.82). This result indicates that although 2-year-olds might use the tool relatively frequently one-time, but at the age of 3-4 tool use is a more robust phenomenon.

With respect to the Irrelevant-Relevant condition, Mann-Whitney test showed that in Session 1 children’s performance was similar (*U* = 162, *p* = 1, *d* = 0), and in Session 2 marginal difference was found (*U* = 108, *p* = .091, *d* = 0.59). With respect to this result, we followed the suggestion of increasing the power of the comparison test without increasing the sample size (Lakens, 2021). Since in our setup the prediction can only have one concrete direction, namely, due to the constraint change a more detailed memory retrieval is required in the relevant situation in contrast to the irrelevant (and not vica versa), a valid option is to apply a directional (one-sided) t-test. The one sided Mann-Whitney test gave significant result (*U* = 108, *p* = .045, *Cohen’s d* = 0.59).

The data that support the findings of this study are openly available in Open Science Foundation at: <https://osf.io/jx4q6/?view_only=ffd5fb77c46c4729bce5b41d6077f38e>
